# Complete Mitochondrial Genomes of *Pentapodus caninus* and *Lethrinus olivaceus* (Spariformes: Nemipteridae and Lethrinidae): Genome Characterization and Phylogenetic Analysis

**DOI:** 10.3390/ani15243526

**Published:** 2025-12-07

**Authors:** Nan Chen, Mingcan Gu, Wenqing Jiang, Lei Xie, Qi Qiao, Jingyi Cen, Yuelei Dong, Songhui Lu, Lei Cui

**Affiliations:** Key Laboratory of Eutrophication and Red Tide Prevention of Guangdong Higher Education Institutes, College of Life Science and Technology, and Southern Marine Science and Engineering Guangdong Laboratory (Zhuhai), Jinan University, Guangzhou 510632, China; chennan@stu2023.jnu.edu.cn (N.C.); gumingcan@stu.jnu.edu.cn (M.G.); jiangwenqing@stu.jnu.edu.cn (W.J.); xl0224@stu2022.jnu.edu.cn (L.X.); qiaoqi@stu2022.jnu.edu.cn (Q.Q.); jingyicen@gmail.com (J.C.); yldong@jnu.edu.cn (Y.D.)

**Keywords:** Nemipteridae, Lethrinidae, gene rearrangement, systematics, Indo-West Pacific, marine fishes

## Abstract

Complete mitochondrial genomes (mitogenomes) are widely utilized molecular resources for phylogenetic studies. Although research on Spariformes mitogenomes has advanced significantly, there is still relatively little information regarding the molecular data and taxonomic placement of the families Nemipteridae and Lethrinidae. We report and annotate the first complete mitogenomes of *Pentapodus caninus* (16,866 bp; Nemipteridae) and *Lethrinus olivaceus* (16,792 bp; Lethrinidae), thereby expanding mitogenomic coverage in two families with limited available genomic data. Both assembled mitogenomes display the canonical vertebrate architecture, comprising 37 functional genes (13 protein-coding genes, 22 tRNAs, and 2 rRNAs) and a control region, with conserved synteny and strand asymmetry (only *ND6* and eight tRNAs are light-strand encoded). While ATG serves as the primary initiation codon for most PCGs, *COX1* employs an alternative GTG start codon. Structural analysis of tRNAs revealed that most sequences adopt the standard cloverleaf conformation, with the exception of *tRNA-Ser^AGY^*, which lacks the dihydrouridine (DHU) arm. A rare tandem duplication of *tRNA-Val* in *Lethrinus* species highlights the structural variability of spariform mitochondrial genomes. Furthermore, phylogenomic reconstruction using the concatenated 13 protein-coding gene dataset recovered Nemipteridae and Sparidae as sister taxa. In this topology, Lethrinidae was identified as the earliest diverging lineage, basal to the Nemipteridae–Sparidae grouping. Our results not only advance our understanding of the origin and evolution of Spariformes, but also provide valuable information for the molecular phylogeny and taxonomy of teleostean species.

## Simple Summary

Mitochondrial DNA, typically characterized by its circular structure and maternal inheritance, provides essential genetic information for reconstructing the evolutionary history of species. We report the first complete mitochondrial genomes of two coastal fishes, *Pentapodus caninus* and *Lethrinus olivaceus*. Phylogenetic analyses using these and published mitochondrial genomes clarify relationships within the spariform fishes: Nemipteridae (threadfin breams) is recovered as sister to Sparidae (porgies), while Lethrinidae (emperors) occupies a more basal position relative to that clade. We also found a rare duplication of a transfer RNA gene in *Lethrinus*, showing that mitochondrial structure can change. These newly characterized mitogenomes facilitate taxonomic distinction and provide robust molecular data for reconstructing the evolutionary history of spariform fishes.

## 1. Introduction

Spariformes has been recognized as a stable percomorph order as a result of higher-level revisions that resolved the demonstrably paraphyletic ‘Perciformes’ sensu lato using expanded molecular datasets [1,2]. The order, comprising Lethrinidae (emperors), Nemipteridae (threadfin breams), and Sparidae (porgies, including former Centracanthidae), contains ecologically pivotal and economically valuable teleosts associated with reefs and soft bottom, distributed from tropical to temperate waters [1,3]. Yet relationships within Spariformes—and their placement relative to closely allied percomorph lineages—remain incompletely resolved. For example, morphology-based hypotheses have variously positioned Nemipteridae as a basal lineage, whereas early molecular studies yielded conflicting arrangements, such as a sister relationship between Lethrinidae and Nemipteridae versus a clade uniting Nemipteridae and Sparidae [4,5,6,7]. Robust resolution is necessary to reconstruct biogeographic history, understand trophic and ecological trait evolution, and refine taxonomic boundaries [8,9].

Within this framework, Lethrinidae and Nemipteridae are key components of Indo-West Pacific coastal fisheries and food security. Many species are widely distributed, targeted by multi-gear artisanal and commercial fleets, and display trophic and morphological convergence that can confound field identifications [10,11]. Reports of geographically structured lineages and suspected cryptic diversity in several genera further underscore the need for authoritative molecular references to support species delimitation, traceability, and enforcement [12]. Mitogenomes provide rich phylogenetic signal beyond single-locus barcodes, including compositional skews (informative for strand-specific mutational regimes), codon-usage bias (reflecting translational and tRNA adaptation), and control-region organization (tandem repeats and conserved sequence blocks tracking lineage demographic or regulatory shifts), all of which illuminate lineage-specific evolutionary dynamics [13]. However, as a single locus, mitochondrial DNA can be subject to introgression or incomplete lineage sorting, meaning the resulting gene tree may not always fully reflect the true species phylogeny. Furthermore, mitogenome-based phylogenetics can be biased by compositional heterogeneity, saturation at third codon positions, and maternal inheritance, motivating careful data partitioning and model selection to assess topological robustness.

To address gaps in mitogenomic resources and improve phylogenetic sampling within Spariformes, particularly in Nemipteridae and Lethrinidae, we focused on two widely distributed species: *Lethrinus olivaceus* Valenciennes, 1830 and *Pentapodus caninus* (Cuvier, 1830). *L. olivaceus* is a large carnivorous lethrinid that inhabits reef-associated habitats across the Indo-West Pacific and represents a key target for local fisheries [14,15,16]. *P. caninus* is distributed across the western Pacific from the South China Sea to the western Pacific islands and occupies nearshore soft-bottom and reef-sand interfaces [15,16]. Species-level identification can be problematic in mixed catches. Lethrinidae has been the subject of extensive research on trophic diversification and species limits using morphology, morphometrics, and mitochondrial markers, revealing repeated ecological shifts and complex lineage structure across ocean basins [8,9,17]. In parallel, recent molecular investigations have focused on Nemipteridae, clarifying relationships among genera and uncovering geographically diversified genetic structure in *Nemipterus* spp. across the Red Sea and Mediterranean [18,19].

Despite their ecological and economic significance, the availability of complete mitochondrial genome resources remains uneven across spariform families. According to an NCBI search (accessed September 2025), complete mitogenomes are publicly available for 32 Sparidae species, whereas Nemipteridae has only nine and Lethrinidae only six, limiting comprehensive, family-spanning comparisons of genome architecture, control-region organization, codon-usage bias, compositional skews, and lineage-specific rate variation. This imbalance constrains the ability to evaluate how general patterns (e.g., convergent trophic evolution, mitogenomic compositional trends) inferred from Sparidae extend to underrepresented families [5,7,20].

This study presents annotated, newly generated complete mitogenomes for *P. caninus* (Nemipteridae) and *L. olivaceus* (Lethrinidae), characterizing their genome architecture, nucleotide composition, protein-coding and RNA genes, and control-region features. By integrating these sequences with published Spariformes mitogenomes, we reconstructed family-level relationships using partitioned maximum-likelihood (ML) and Bayesian inference (BI) analyses. The inclusion of two previously underrepresented families enhances mitogenomic coverage across Spariformes, thereby refining phylogenetic resolution and providing genomic references for studies of molecular identification, population genetics, and molecular evolution. Furthermore, this study uncovers a rare *tRNA-Val* tandem duplication in *L. olivaceus*, revealing a lineage-specific structural rearrangement within Lethrinidae.

## 2. Materials and Methods

### 2.1. Sample Collection and Genomic DNA Isolation

*P. caninus* and *L. olivaceus* specimens were collected from the South China Sea which (14°52′ N–17°69′ N, 111°22′ E–115°69′ E) is located at the edge of the Western Pacific Ocean in March 2025. All procedures in this study complied with the guidelines of the IUCN Red List, involving no endangered or protected species, and received approval from the Jinan University Laboratory Animal Welfare and Ethics Committee. Specimen identification was conducted based on morphological traits, after which samples were preserved in 95% ethanol and cryopreserved at −80 °C prior to analysis. We isolated total genomic DNA from dorsal muscle biopsies utilizing the Animal Tissue Genomic DNA Extraction Kit (SangonBiotech, Shanghai, China), strictly adhering to the manufacturer’s protocol. Subsequently, the purified DNA served as the template for Polymerase Chain Reaction (PCR) to amplify the complete mitochondrial genomes of *P. caninus* and *L. olivaceus* [21,22].

The newly obtained mitogenome sequences of the *P. caninus* and *L. olivaceus* were submitted to the GenBank database under the accession number PV872034 and PV872036, respectively.

### 2.2. Amplification and Sequencing of Mitochondrial Genomes

Primers for amplifying the complete mitochondrial genomes of *P. caninus* and *L. olivaceus* were designed by aligning reference sequences from *Pentapodus setosus* (Valenciennes, 1830) (NC_086456.1) and *Lethrinus laticaudis* Alleyne & Macleay, 1877 (NC_030353.1), as detailed in Tables S1 and S2 [21]. We performed PCR assays using the Premix LA Taq system (Takara, Dalian, China) containing LA Taq DNA polymerase. The thermal cycling profile was programmed as follows: an initial denaturation at 95 °C for 1 min; 35 cycles comprising denaturation (95 °C, 20 s), annealing (55 °C, 45 s), and extension (72 °C, 1–3 min based on fragment size). Final amplicons were sequenced using a 3730XL DNA Analyzer at the Beijing Genomics Institute (Shenzhen, China).

### 2.3. Mitochondrial Genome Assembly

In order to obtain the final complete sequence, the obtained sequenced fragments were assembled through the program Contig Assembly within the SnapGene software (www.snapgene.com, accessed on 1 June 2025) and then manually checked. Gene annotation, including 13 protein-coding genes (PCGs), 22 tRNAs, and two rRNAs, was performed using the MITOS web server [23], with gene boundaries and orientations subsequently cross-validated via the MitoFish pipeline [24]. To ensure accuracy, PCG identities were confirmed by BLAST+ 2.16.0 searches against available Spariformes mitogenomes in the NCBI database [25]. A visual representation of *P. caninus* and *L. olivaceus* mitogenome was created by the CGView online program (https://www.bioinformatics.org/cgview/download.html, accessed on 10 June 2025).

We predicted the secondary structures of tRNA genes and the light-strand replication origin using RNAstructure [26]. Nucleotide composition and codon usage metrics were calculated in MEGA 11.0 [27]. For Relative Synonymous Codon Usage (RSCU) calculations, we excluded all stop codons to ensure reading frame consistency and calculation accuracy. Nucleotide asymmetries were quantified using the standard skew formulas: AT-skew = (A − T) / (A + T) and GC-skew = (G − C) / (G + C).”

### 2.4. Comparative Phylogenetic Analysis

To elucidate the evolutionary placement of *P. caninus* and *L. olivaceus* within Spariformes, we analyzed a dataset comprising 35 teleost mitogenomes. The ingroup included 33 Spariformes species representing three families: Nemipteridae (11), Sparidae (15), and Lethrinidae (7). *Perca fluviatilis* Linnaeus, 1758 (Percidae, NC_026313) and *Epinephelus coioides* (Hamilton, 1822) (Serranidae, NC_011111) were selected as outgroups. All sequences were retrieved from GenBank.

The 13 protein-coding genes (PCGs) were extracted and aligned using MAFFT, followed by codon-aware refinement in MACSE [28]. The resulting alignments were concatenated in the gene order: *ND1*-*ND2*-*COX1*-*COX2*-*ATP8*-*ATP6*-*COX3*-*ND3*-*ND4L*-*ND4*-*ND5*-*ND6*-*CYTB*. We employed ModelFinder to identify the optimal partitioning scheme and substitution models based on the lowest Bayesian Information Criterion (BIC) scores [29] (Table S3). Phylogenetic reconstruction was performed using both ML and BI approaches. The ML tree was inferred in IQ-TREE [30] with 1000 bootstrap replicates to assess nodal support [30]. The BI phylogenetic analysis was conducted in MrBayes under the GTR+G substitution model. We performed two independent analyses, each consisting of four simultaneous chains run for 10,000,000 generations [31]. Trees were sampled every 100 generations, with the initial 25% discarded as burn-in. Stationarity and convergence were verified by ensuring that the average standard deviation of split frequencies fell below 0.01 and that effective sample size (ESS) values for all parameters exceeded 200. All of the above were available on the PhyloSuite platform [32]. Final tree topologies were visualized and annotated using iTOL [33].

## 3. Results

### 3.1. Mitogenomic Architecture and Base Composition

**Figure 1:**
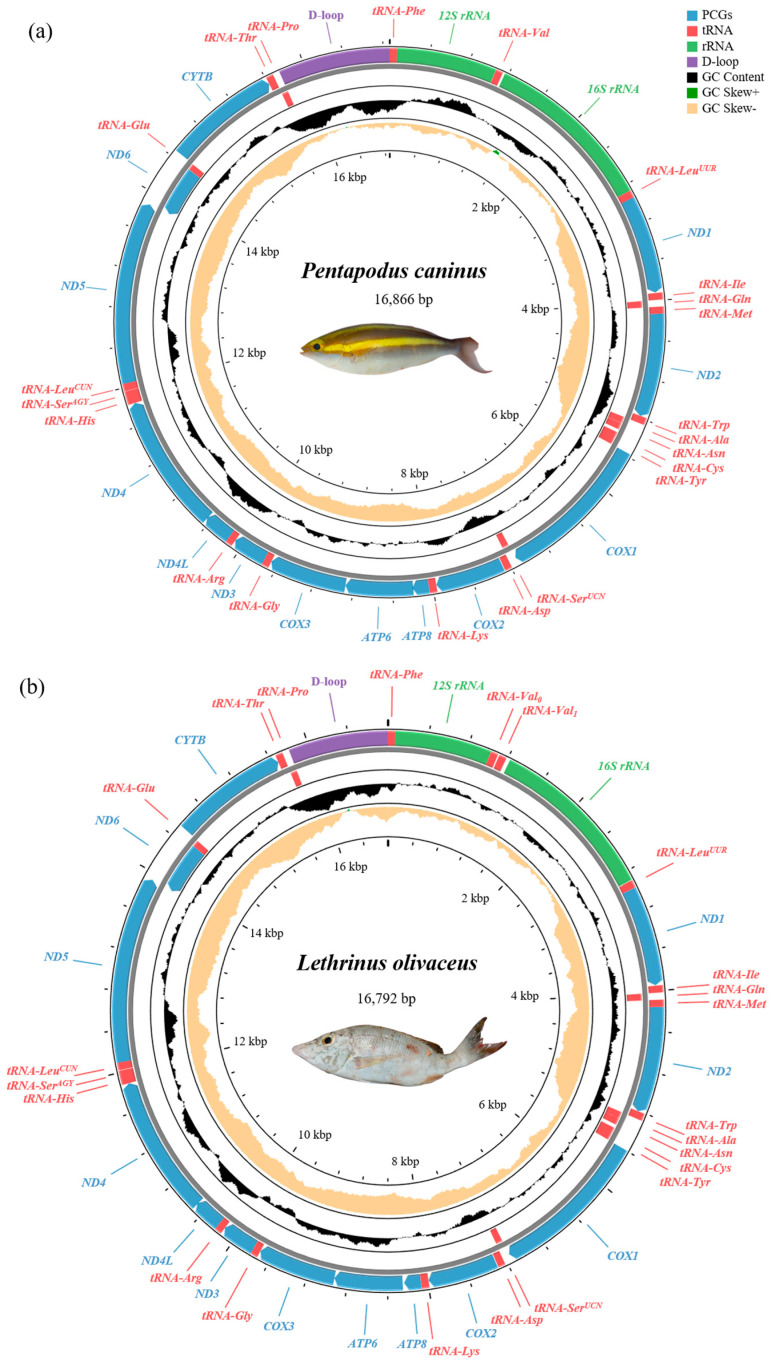
Circular genome visualizations for *P. caninus* (**a**) and *L. olivaceus* (**b**). Genes encoded on the heavy strand are plotted on the outer circle, whereas those on the light strand are positioned on the inner circle.

The complete circular mitogenomes of *P. caninus* and *L. olivaceus* spanned 16,866 bp and 16,792 bp, respectively. Both genomes displayed the canonical vertebrate mitochondrial architecture, consisting of 37 functional genes—including 13 PCGs, 22 tRNAs, and two rRNAs—alongside a major non-coding control region (D-loop) (Table 1 and Table 2; Figure 1) [34]. Gene order conforms to the canonical teleost arrangement, and no gene rearrangements were detected [35]. *ND6* and eight tRNA genes (*tRNA-Glu*, *tRNA-Pro*, *tRNA-Gln*, *tRNA-Ala*, *tRNA-Asn*, *tRNA-Cys*, *tRNA-Tyr*, *tRNA-Ser*) are encoded on the light strand (L-strand), whereas the remaining genes are located on the heavy strand (H-strand) (Figure 1) [36]. We detected 14 intergenic spacer regions (totaling 85 bp) and 10 overlaps (29 bp) in *P. caninus*, and 15 spacers (135 bp) together with 9 overlaps (19 bp) in *L. olivaceus*.

An overall bias toward A+T content was observed in both species. In *P. caninus*, the nucleotide distribution was A (28.1%), T (27.5%), G (16.9%), and C (27.5%), resulting in a total A+T content of 55.6%. Analysis of strand asymmetry revealed a positive AT-skew (0.010) and a negative GC-skew (−0.238), reflecting a slight preference for Adenine over Thymine and Cytosine over Guanine. Similarly, *L. olivaceus* presented an A+T content of 52.6% (A: 26.6%, T: 26.0%, G: 17.2%, C: 29.7%), with comparable skew metrics (AT-skew = 0.013; GC-skew = −0.265).”

### 3.2. Analysis of Protein-Coding Sequences

The combined length of the 13 PCGs is 11,433 bp in *P. caninus* and 11,430 bp in *L. olivaceus*, accounting for approximately 67.8% and 68.1% of the total genome size, respectively (Table 1 and Table 2). In terms of gene size, *ATP8* (168 bp) is the smallest element, while *ND5* (1839 bp) represents the largest coding region in both genomes. The transcriptional orientation is conserved: *ND6* is the sole PCG encoded on the Light (L) strand, with the remaining twelve genes situated on the Heavy (H) strand.

Initiation codons are predominantly ATG across both mitogenomes; notably, *COX1* initiates with GTG. Termination codons include both complete (TAA/TAG) and incomplete forms. According to our annotations, *ND1*-*ND3*, *ND4L*, *ND5*, *ND6*, *COX1*, *COX3*, *ATP8*, and *ATP6* terminate with complete codons, whereas *COX2*, *ND4*, and *CYTB* display incomplete stop codons (Table 1 and Table 2).

Across PCGs, base composition is modestly AT-biased in both species. In *P. caninus*, A+T ranges from 47.8% (*ND4L*) to 57.1% (*ATP8*), with a mean across PCGs of 55.6%; in *L. olivaceus*, A+T ranges from 44.5% (*ND4L*) to 55.4% (*ATP8*), averaging 52.6% (Tables S4 and S5). Strand-asymmetric base skews are apparent at the gene level: most PCGs show negative GC-skew, whereas *ND6*, encoded on the opposite strand, shows the opposite tendency.

### 3.3. Codon Usage Patterns and Amino Acid Frequency

The amino acid composition of the 13 PCGs was analyzed for both species. In total, the PCGs of *P. caninus* and *L. olivaceus* encode 3800 and 3799 amino acids, respectively (excluding stop codons). The amino acid frequency distributions were highly similar between the two species. Leucine is the most abundant amino acid (16.5% in *P. caninus*; 17.4% in *L. olivaceus*), followed by alanine and threonine, whereas cysteine is the least frequent (0.8% and 0.6%, respectively) (Table S7).

**Figure 2:**
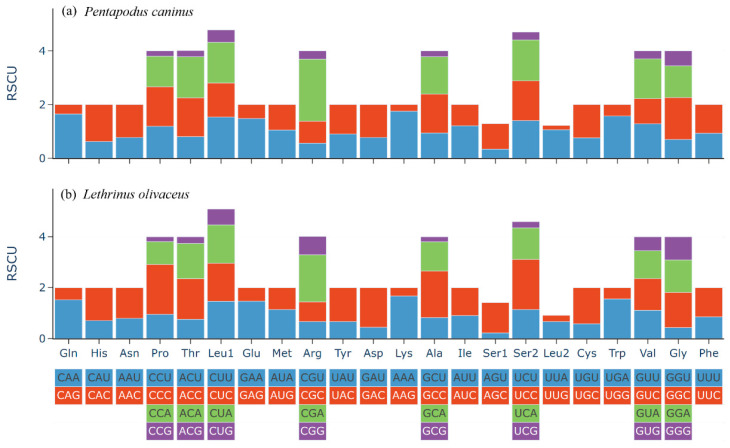
Analysis of codon usage bias in mitochondrial PCGs. Charts display codon frequencies and relative synonymous codon usage (RSCU) values for *P. caninus* (**a**) and *L*. *olivaceus* (**b**).

To investigate codon-usage bias, RSCU values were calculated (Figure 2). A clear pattern of nonuniform codon usage was observed. The most notable trend is a strong bias against G at third codon positions across most synonymous families; within fourfold-degenerate families in particular, G-ending codons were the most underrepresented (RSCU < 0.3). For example, in both species, GCG (Ala), CCG (Pro), and ACG (Thr) are rare. By contrast, C- or A-ending codons are often preferred. In *L. olivaceus*, CCC (Pro, RSCU = 1.96) and UCC (Ser, RSCU = 1.97) are highly favored; in *P. caninus*, CGA (Arg, RSCU = 2.31) and AAA (Lys, RSCU = 1.75) show strong positive bias.

### 3.4. Characterization of RNA Genes

#### 3.4.1. rRNA Genes

The mitogenomes of *P. caninus* and *L. olivaceus* contain the standard set of two ribosomal RNA genes. The large subunit (*16S rRNA*) is 1705 bp and 1669 bp long, and the small subunit (*12S rRNA*) is 997 bp and 955 bp long in *P. caninus* and *L. olivaceus*, respectively. The two ribosomal RNA genes are encoded on the H-strand and are interspersed with *tRNA-Val*. Notably, while *P. caninus* possesses a single copy of *tRNA-Val*, *L. olivaceus* exhibits a tandem duplication of this gene. Specifically, the *12S rRNA* is flanked by *tRNA-Phe* and *tRNA-Val*, whereas the *16S rRNA* is positioned between *tRNA-Val* and *tRNA-Leu* (Table 1 and Table 2).

#### 3.4.2. General Features of tRNA Genes

All 22 typical tRNA genes are identified, with total lengths of 1561 bp in *P. caninus* and 1638 bp in *L. olivaceus*. Individual tRNAs range from 67 to 76 bp (Table 1). Fourteen tRNAs are encoded on the H-strand and eight on the L-strand. Predicted anticodons match the standard vertebrate mitochondrial genetic code, with no lineage-specific substitutions (Table 1).

#### 3.4.3. Tandem Duplication of tRNA-Val in *L. olivaceus*

**Figure 3:**
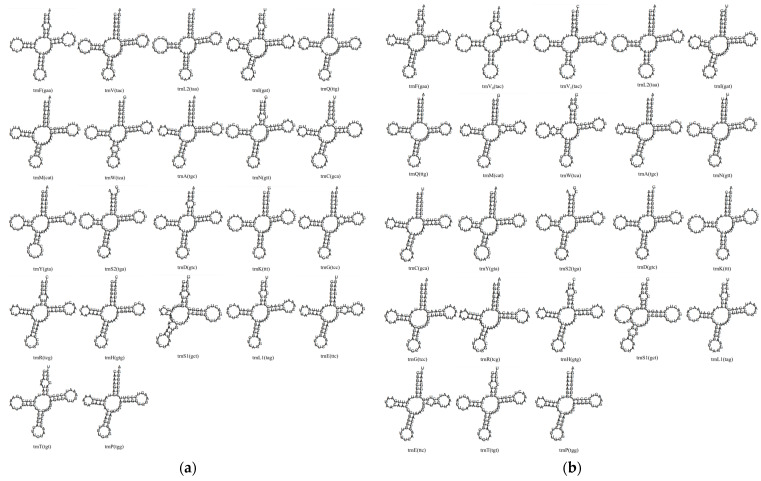
Putative secondary structures for the complete set of 22 mitochondrial tRNAs in *P. caninus* (**a**) and *L. olivaceus* (**b**).

A notable feature in the mitogenome of *L. olivaceus* is a tandem duplication of the *tRNA-Val* gene located between the *12S* and *16S rRNA* genes. The two copies, designated *tRNA-Val*_0_ and *tRNA-Val*_1_, are separated by a 13 bp non-coding spacer and are encoded on the same strand (Table 2). Both copies retain the canonical valine anticodon (UAC) and fold into nearly identical, stable cloverleaf secondary structures (Figure 3). Only three single-nucleotide differences are observed between them, and their structural integrity suggests that both copies are likely functional. The presence of this duplication is further confirmed by targeted PCR amplification and subsequent Sanger sequencing across the entire locus (Table S1). A tandem duplication of the *tRNA-Val* gene within the *12S*–*16S rRNA* region represents a rare but recurrent structural variation within Lethrinidae. According to comparative mitogenomic data, only three species, *L. olivaceus*, *Lethrinus nebulosus* (Fabricius, 1775), and *Lethrinus obsoletus* (Fabricius, 1775), exhibit an extended *12S*-*tRNA-Val*-*16S* region (165–202 bp) and confirmed *tRNA-Val* duplication, whereas the remaining lethrinid species possess a typical short interval (72–76 bp) with a single copy (Table S6).

#### 3.4.4. tRNA Secondary Structures

Predicted secondary structures for all 22 tRNAs in both species are cloverleaf forms except *tRNA-Ser^AGY^*, which lacks the dihydrouridine (DHU) arm (Figure 3). G-U wobble pairs are present in stem regions (35 in *P. caninus* and 35 in *L. olivaceus*), and no additional noncanonical mismatches were detected.

### 3.5. Characteristics of Control Region

The control region represents the primary non-coding sequence in both *P. caninus* and *L. olivaceus*. Consistent with the conserved vertebrate mitogenome architecture, this region is situated between the *tRNA-Pro* and *tRNA-Phe* genes. The control region is 1114 bp in *P. caninus* and 984 bp in *L. olivaceus*. This region shows a pronounced AT bias (62.5% in *P. caninus*; 60.0% in *L. olivaceus*) and a negative GC-skew (−0.062 and −0.178, respectively), reflecting mutational pressures typical of the heavy strand.

At the 5’ end (*tRNA-Pro* side), a termination-associated sequence (TAS)-like motif containing a TACAT core is present (*P. caninus*: “…TTATACATGGTG…”); in *L. olivaceus*, a TACACT variant is observed (“…CAGGTACACTCAT…”). Toward the 3’ end (*tRNA-Phe* side), both species exhibit a conserved-sequence-block cluster comprising a CSB-1-like element defined by “TAAAC” followed by a C-rich tract immediately adjacent to a CSB-2-like poly-C/G box (for example, “TAAACCCCCCTACCCCCCTA”), with an AAAC/AAACA short motif downstream as a CSB-3-like candidate. Other conserved blocks, such as CSB-D/E/F, are not clearly identifiable [37].

### 3.6. Phylogenetic Relationships

**Figure 4:**
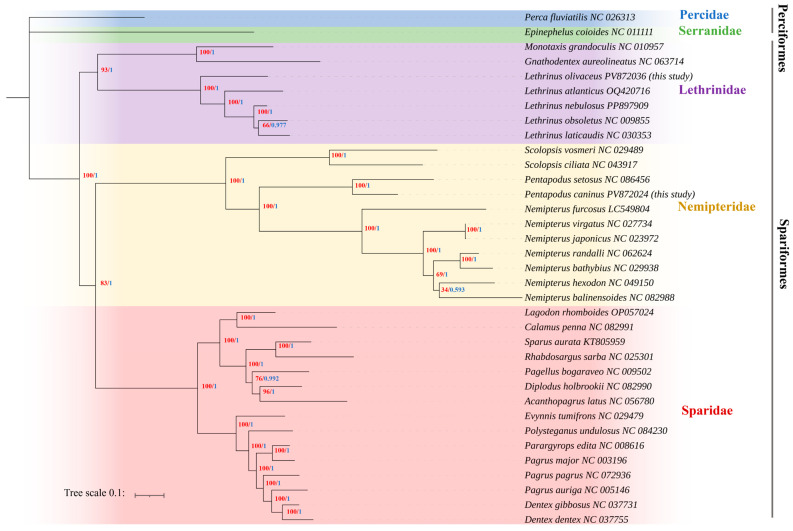
Phylogenetic reconstruction of Spariformes relationships. The tree topology was inferred from the concatenated dataset of 13 PCGs using Maximum Likelihood (ML) and Bayesian Inference (BI). Nodal support is indicated by numbers on branches: red values represent ML bootstrap percentages, while blue values denote Bayesian posterior probabilities. *Perca fluviatilis* and *Epinephelus coioides* were included as outgroups.

To clarify the phylogenetic positions of the two newly sequenced species, *P. caninus* and *L. olivaceus*, and to refine interfamilial relationships within Spariformes, we infer ML and BI trees from the concatenated nucleotide sequences of 13 mitochondrial protein-coding genes across 35 ingroup taxa, using *Perca fluviatilis* and *Epinephelus coioides* as outgroups (Figure 4).

*L. olivaceus* is recovered as the earliest-diverging lineage among the sampled *Lethrinus* species. *Monotaxis grandoculis* (Fabricius, 1775) and *Gnathodentex aureolineatus* (Lacepède, 1802) fall just outside the *Lethrinus* radiation, and internal *Lethrinus* groupings show short terminal branches with strong support. The sequencing of *L*. *olivaceus* adds to the mitogenomic representation of the genus, corroborating its phylogenetic placement within Lethrinidae. In Nemipteridae, *Pentapodus caninus* clusters with *P*. *setosus*, recovering a monophyletic *Pentapodus* lineage. This lineage forms a sister group to *Scolopsis*, while *Nemipterus furcosus* (Valenciennes, 1830) appears genetically divergent from other sampled Nemipterus species, suggesting potential generic-level distinctiveness that warrants further investigation. Within Sparidae, two strongly supported assemblages are recovered: a (*Dentex*, *Pagrus*) cluster and a (*Sparus*, *Rhabdosargus*, *Diplodus*) cluster.

The ML and BI analyses yield an identical overall topology with uniformly high nodal support, recovering unequivocal monophyly of the three sampled families and the backbone arrangement (Lethrinidae, (Nemipteridae, Sparidae)).

## 4. Discussion

The overall lengths (16,866 bp in *P. caninus*; 16,792 bp in *L. olivaceus*) and the standard complement and arrangement of 37 mitochondrial genes conform to the canonical teleost pattern, indicating absence of large-scale structural innovation in these two newly sequenced genomes. The counts and total sizes of intergenic spacers and overlaps fall within the range commonly reported for percomorph mitogenomes and are consistent with a compact genome architecture shaped by the tRNA-punctuation model [35,36]. The positive AT-skew combined with negative GC-skew in both whole mitogenomes reflects strand-asymmetric mutation patterns linked to replication-associated deamination and differential single-strand exposure during transcription/replication in vertebrate mitochondrial DNA [21,36]. These compositional skews and overall proportions are also consistent with patterns reported for Spariformes and related percomorph lineages [3,20,35], supporting conserved mutational or regulatory constraints among these taxa.

The uniformity of protein-coding gene lengths (e.g., shortest *ATP8* and longest *ND5*) and strand distribution (*ND6* on the L-strand, remaining PCGs on the H-strand) matches previously described teleost gene organizations. The use of GTG as the initiation codon for *COX1* agrees with frequent alternative starts documented for teleost *COX1* [13], suggesting conserved translational flexibility rather than a lineage-specific innovation. The mixture of complete and incomplete stop codons (*COX2*, *ND4*, *CYTB*) follows the well-known pattern whereby incomplete terminal codons are completed post-transcriptionally by polyadenylation [38]. Overall start/stop codon usage falls within the range reported for other Spariformes and related percomorph lineages [35], implying no unusual selection on translational initiation or termination signals. The contrasting GC-skew of *ND6* relative to H-strand PCGs reflects the expected effect of strand location under replication-associated mutational bias in teleost mitogenomes [39]. This pattern reinforces the interpretation that compositional asymmetry reflects replication-associated mutational/repair processes tied to strand exposure, rather than locus-specific adaptive optimization.

Similarity in amino acid frequency profiles between the two species, with Leu, Ala, and Thr dominant and Cys rare, mirrors common teleost mitochondrial trends and suggests broadly conserved functional constraints on mitochondrially encoded proteins. The pronounced underrepresentation of G-ending codons (RSCU < 0.3 in multiple four-fold-degenerate families) and preferential use of A- or C-ending codons reflect underlying nucleotide skews at third positions. The avoidance of third-position G corresponds to the negative GC-skew of most H-strand genes and is consistent with asymmetric mutational pressure against guanine during replication [13]. Thus, codon usage patterns in *P. caninus* and *L. olivaceus* appear characteristic of teleost mitogenomes, shaped predominantly by mutational biases rather than by strong translational selection, and are comparable to those reported for other Spariformes species [3,20].

The lengths and arrangement of *12S* and *16S rRNA* genes, separated by *tRNA-Val*, match the typical vertebrate organization. The tandem duplication of *tRNA-Val* in *L. olivaceus* (two intact copies separated by a 13 bp spacer) represents a structural variant documented in a subset of Lethrinidae (*L. olivaceus*, *L. nebulosus*, *L. obsoletus*) showing expanded *12S*–*tRNA-Val*–*16S* intervals, whereas other Lethrinidae species retain a single-copy compact configuration (Table S6). Sequence comparisons in related species (one canonical plus one divergent or variant copy) suggest multiple processes—full tandem duplication, fragmental duplication, and subsequent sequence divergence or degeneration—have contributed to heterogeneity in this locus. Preservation of functional anticodons and predicted stable cloverleaf structures in both *L. olivaceus* copies supports potential retained functionality rather than incipient pseudogenization. Placement at the canonical rRNA junction and maintenance of structural integrity are consistent with a tandem duplication–random loss mechanism recognized for mitochondrial gene rearrangements [34,40]. Under the tRNA punctuation model, duplicated cleavage signals may be selectively neutral if transcript processing efficiency is not impaired [41]. Comparable tRNA duplications (including pseudogenized derivatives) in other teleost lineages (e.g., Labridae: *Chlorurus sordidus* (Fabricius, 1775)) [42,43] illustrate that such events, though infrequent, recur across Eupercaria and contribute sporadically to mitogenomic structural diversity [43,44]. The absence of noncanonical mismatches beyond expected G–U wobble pairs and the conserved loss of the DHU arm in *tRNA-Ser^AGY^*—a characteristic vertebrate feature [45,46]—indicate strong functional constraints on tRNA secondary structure despite occasional duplication events.

Control-region lengths and marked AT bias with negative GC-skew values further reflect mutational pressures typical of the heavy strand, reinforcing shared replication/transcription dynamics in these mitogenomes. Detection of a TAS-like motif containing a TACAT core (variant TACACT in *L. olivaceus*) at the 5′ end and a downstream cluster of CSB-1-like (“TAAAC”), adjacent C-rich tract (CSB-2-like poly-C/G box), and an AAAC/AAACA short element (CSB-3-like candidate) at the 3′ end recapitulate canonical teleost control-region organization. The inability to clearly delimit additional conserved blocks (CSB-D/E/F) may reflect elevated sequence divergence in these lineages [37]. Overall TAS–CSB arrangement and compositional patterns indicate conserved regulatory architecture across Spariformes, with no evidence for lineage-specific acquisition or loss of major functional motifs.

The inclusion of the new complete mitogenomes for *P*. *caninus* and *L*. *olivaceus* enhances phylogenetic resolution within the Spariformes, providing robust support for relationships among major families. The recovery of *L*. *olivaceus* as the sister lineage to all other sampled *Lethrinus* species provides critical insight into the basal diversification of the genus. The clustering of *P. caninus* with *P. setosus* confirms a distinct *Pentapodus* clade separate from *Scolopsis* and the *Nemipterus* core assemblage. Recovery of two well-supported Sparidae subclusters (*Dentex*, *Pagrus*) and (*Sparus*, *Rhabdosargus*, *Diplodus*) agrees with prior mitogenomic topologies. Whereas previous work reported negligible bootstrap support (bootstrap = 36) for the arrangement ((Lethrinidae, Nemipteridae), Sparidae) [35], our analyses strongly resolve the topology as (Lethrinidae, (Nemipteridae, Sparidae)), corroborating a closer sister relationship between Nemipteridae and Sparidae. This strengthened support may reflect expanded taxon sampling and full PCG concatenation under both ML and BI frameworks. Further progress will benefit from denser taxon sampling (particularly unsampled genera and species) and incorporation of independent nuclear datasets and site-heterogeneous models to more fully test backbone relationships within Spariformes.

## 5. Conclusions

This study presents two new complete mitochondrial genomes for *L*. *olivaceus* and *P*. *caninus*, expanding the mitogenomic representation of Lethrinidae and Nemipteridae. Both mitogenomes conform to the canonical teleost organization (13 PCGs, 22 tRNAs, 2 rRNAs, and a control region) with conserved gene order and overall AT bias. A rare tandem duplication of the *tRNA-Val* gene was identified in *Lethrinus olivaceus* and related species, highlighting structural flexibility and lineage-specific rearrangements within Lethrinidae mitogenomes. The primary contribution of this work is the clarification of major phylogenetic uncertainties. ML and BI analyses of concatenated PCGs recover an identical, well-supported topology that confirms the monophyly of Lethrinidae, Nemipteridae, and Sparidae and resolves the backbone as (Lethrinidae, (Nemipteridae, Sparidae)). This study provides a revised phylogenetic framework for Spariformes, which can inform future comparative genomic and systematic research on this fish order. The genomic data generated provide valuable resources for taxonomic distinction and population genetic studies. However, future work incorporating nuclear genome-wide data is essential to test these mitochondrial hypotheses and fully resolve the evolutionary history of this group.

## Figures and Tables

**Table 1:** Organization and genomic features of the *P. caninus* mitogenome. “H” and “L” denote genes located on the heavy and light strands, respectively.

Feature	Position		Size	Strand	Spacer (+) /Overlap (−)	Start/Stop Codon	AntiCodon
	Start	End					
*tRNA-Phe*	1	71	71	H	0	-	GAA
*12S rRNA*	72	1068	997	H	2	-	-
*tRNA-Val*	1071	1142	72	H	22	-	TAC
*16S rRNA*	1165	2869	1705	H	1	-	-
*tRNA-Leu^UUR^*	2871	2944	74	H	0	-	TAA
*ND1*	2945	3919	975	H	4	ATG/TAA	-
*tRNA-Ile*	3924	3993	70	H	−1	-	GAT
*tRNA-Gln*	3993	4063	71	L	−1	-	TTG
*tRNA-Met*	4063	4132	70	H	0	-	CAT
*ND2*	4133	5179	1047	H	−1	ATG/TAA	-
*tRNA-Trp*	5179	5250	72	H	0	-	TCA
*tRNA-Ala*	5251	5319	69	L	2	-	TGC
*tRNA-Asn*	5322	5394	73	L	34	-	GTT
*tRNA-Cys*	5429	5497	69	L	0	-	GCA
*tRNA-Tyr*	5498	5568	71	L	1	-	GTA
*COX1*	5570	7120	1551	H	1	GTG/TAA	-
*tRNA-Ser^UCN^*	7122	7192	71	L	2	-	TGA
*tRNA-Asp*	7195	7266	72	H	7	-	GTC
*COX2*	7274	7964	691	H	0	ATG/T--	-
*tRNA-Lys*	7965	8039	75	H	1	-	TTT
*ATP8*	8041	8208	168	H	−10	ATG/TAA	-
*ATP6*	8199	8882	684	H	−1	ATG/TAA	-
*COX3*	8882	9667	786	H	−1	ATG/TAA	-
*tRNA-Gly*	9667	9737	71	H	0	-	TCC
*ND3*	9738	10,088	351	H	−2	ATG/TAG	-
*tRNA-Arg*	10,087	10,155	69	H	0	-	TCG
*ND4L*	10,156	10,452	297	H	−7	ATG/TAA	-
*ND4*	10,446	11,826	1381	H	0	ATG/T--	-
*tRNA-His*	11,827	11,895	69	H	0	-	GTG
*tRNA-Ser^AGY^*	11,896	11,962	67	H	3	-	GCT
*tRNA-Leu^CUN^*	11,966	12,038	73	H	0	-	TAG
*ND5*	12,039	13,877	1839	H	−4	ATG/TAA	-
*ND6*	13,874	14,395	522	L	1	ATG/TAA	-
*tRNA-Glu*	14,397	14,465	69	L	4	-	TTC
*CYTB*	14,470	15,610	1141	H	0	ATG/T--	-
*tRNA-Thr*	15,611	15,684	74	H	−1	-	TGT
*tRNA-Pro*	15,684	15,752	69	L	1	-	TGG
D-loop	15,753	16,866	1114	H	0	-	-

**Table 2:** Organization and genomic features of the *L. olivaceus* mitogenome. “H” and “L” denote genes located on the heavy and light strands, respectively.

Feature	Position		Size	Strand	Spacer (+) /Overlap (−)	Start/Stop Codon	AntiCodon
	Start	End					
*tRNA-Phe*	1	68	68	H	0	-	GAA
*12S rRNA*	69	1023	955	H	1	-	-
*tRNA-Val_0_*	1025	1098	74	H	13	-	TAC
*tRNA-Val_1_*	1112	1186	75	H	39		TAC
*16S rRNA*	1226	2894	1669	H	5	-	-
*tRNA-Leu^UUR^*	2900	2973	74	H	0	-	TAA
*ND1*	2974	3945	972	H	4	ATG/TAG	-
*tRNA-Ile*	3950	4019	70	H	−1	-	GAT
*tRNA-Gln*	4019	4089	71	L	−1	-	TTG
*tRNA-Met*	4089	4158	70	H	0	-	CAT
*ND2*	4159	5205	1047	H	−1	ATG/TAA	-
*tRNA-Trp*	5205	5277	73	H	0	-	TCA
*tRNA-Ala*	5278	5346	69	L	1	-	TGC
*tRNA-Asn*	5348	5420	73	L	37	-	GTT
*tRNA-Cys*	5458	5526	69	L	0	-	GCA
*tRNA-Tyr*	5527	5596	70	L	1	-	GTA
*COX1*	5598	7148	1551	H	1	GTG/TAG	-
*tRNA-Ser^UCN^*	7150	7220	71	L	3	-	TGA
*tRNA-Asp*	7224	7295	72	H	7	-	GTC
*COX2*	7303	7993	691	H	0	ATG/T--	-
*tRNA-Lys*	7994	8069	76	H	1	-	TTT
*ATP8*	8071	8238	168	H	13	ATG/TAA	-
*ATP6*	8252	8935	684	H	−1	ATG/TAA	-
*COX3*	8935	9720	786	H	−1	ATG/TAA	-
*tRNA-Gly*	9720	9790	71	H	0	-	TCC
*ND3*	9791	10,141	351	H	−2	ATG/TAG	-
*tRNA-Arg*	10,140	10,208	69	H	0	-	TCG
*ND4L*	10,209	10,505	297	H	−7	ATG/TAA	-
*ND4*	10,499	11,879	1381	H	0	ATG/T--	-
*tRNA-His*	11,880	11,948	69	H	0	-	GTG
*tRNA-Ser^AGY^*	11,949	12,018	70	H	5	-	GCT
*tRNA-Leu^CUN^*	12,024	12,096	73	H	0	-	TAG
*ND5*	12,097	13,935	1839	H	−4	ATG/TAG	-
*ND6*	13,932	14,453	522	L	0	ATG/TAG	-
*tRNA-Glu*	14,454	14,522	69	L	4	-	TTC
*CYTB*	14,527	15,667	1141	H	0	ATG/T--	-
*tRNA-Thr*	15,668	15,740	73	H	−1	-	TGT
*tRNA-Pro*	15,740	15,808	69	L	1	-	TGG
*D-loop*	15,809	16,792	984	H	0	-	-

## Data Availability

The original contributions presented in this study are included in the article/Supplementary Materials. Further inquiries can be directed to the corresponding authors.

## Supplementary Materials

The following supporting information can be downloaded at https://www.mdpi.com/article/10.3390/ani15243526/s1. Table S1: Primer pairs used for PCR amplification of the mitochondrial genomes of *Pentapodus caninus* and *Lethrinus olivaceus*; Table S2: List of mitochondrial genomes from Spariformes and Perciformes species used in this study, with *Perca fluviatilis* and *Epinephelus coioides* included as the outgroups; Table S3: Merged partition scheme and best-fit nucleotide substitution models inferred by ModelFinder for mitochondrial protein-coding genes (PCGs) of 35 fishes’ species; Table S4: Nucleotide composition of the mitochondrial PCGs of *Pentapodus caninus*; Table S5: Nucleotide composition of the mitochondrial PCGs of *Lethrinus olivaceus*; Table S6: Presence or absence of *tRNA-Val* gene duplication in the *12S–tRNA-Val–16S* region across representative Spariformes mitogenomes; Table S7: Codon frequencies and relative synonymous codon usage (RSCU) of the mitochondrial PCGs of fish species of Percoidei.

## Abbreviations

The following abbreviations are used in this manuscript:

PCGs	Protein-coding genes
tRNA	Transfer RNA
rRNA	RibosomalRNA
RSCU	Relative synonymous codon usage
ML	Maximum likelihood
BI	Bayesian inference
DHU	Dihydrouridine
D-loop	Non-coding control region
TAS	Termination-associated sequence
