# Supplementary material for: Complete Mitochondrial Genomes of Pentapodus caninus and Lethrinus olivaceus (Spariformes: Nemipteridae and Lethrinidae): Genome Characterization and Phylogenetic Analysis
Source: Animals (Basel). 2025 Dec 7;15(24):3526. doi: 10.3390/ani15243526 (PMC12729901; doi:10.3390/ani15243526)
Supplement: Supplementary file 1 [file animals-15-03526-s001.zip › animals-4006075-supplementary.pdf]

SUPPLIMENTARY DATA

Table S1. Primer pairs used for PCR amplification of the mitochondrial genomes of *Pentapodus caninus* and *Lethrinus olivaceus*.

Primer*	Sequences (*P. caninus*) 5'→3'	Sequences (*L. olivaceus*) 5'→3'
1F	TTGGTCCTGGCTTTACTGTCAAC	AGCACAAAGGTTTGGTCCTGGC
1R	AGGGCAAGTGATTGCGCTAC	GTTAAGGAGAGGATTTGAACCTCTG
2F	AGGCAAGTCGTAACATGGTAAGC	TGAAACCGGCCCTGAAGCGC
2R	TGCTGCTGTGGCTTGTGTTAG	CCAGAATGGAGTACACTGCTAGGC
3F	TTCTAAGCCTCGCCTGTTTACC	AGACGAGAAGACCCTATGGAGC
3R	CTAAGAGTTTGTAGGATCGAAGCC	GCACTGAGAGTTTTGATCTCTCCGG
4F	CAAGCTCACACTGACTTCTTGC	TGACACCTCTCCCTTCCTATCG
4R	AACCGAGTTGTGCGGGATG	ATGGTGGGCTCATACGATGAACC
5F	TTAGCACTAGCTACAACCACCG	AGCATCCTCAGGTGTAGAAG
5R	GTGATTCCTGTAGGTGGTCAGCAG	GGTTGGGAGGCACCATTCTTTAGC
6F	CCTTGATCCTGACCATGGTATTAAGC	GGAGCAAACCATAGCTTTATACC
6R	ATGGGAGGCATAGCTGCTAC	GGCCCATCAGAGCTTGTCCCCGTAGG
7F	GCACATGGACTAACCTCTTCCG	CCACACAAGCACTGAACCTGTG
7R	GCCAAAGTTTACATCTCGGCAG	TCTGGGTTAAAGCACATGTGGC
8F	GCCTAGACAATGAACAGGACATCCG	AAGACATGGACCTAACCTTCC
8R	CCATTAACTTATGCAAGCGTCA	GAATACTAGCTTTGGGAGTTAGTGG
9F	CTTTCTCTTCAGTGGCACACATCTG	GGCCTATACTATGGGTCCTACCTC
9R	TATGAAGCACCGCCAAGTCC	ATCACTGCTGAAGTTCCCTTGG

*Note: *tRNA-Val* region containing tandem duplication was PCR-amplified and Sanger-sequenced using the following primer pair:
F: 5'-TAGTAAGCACAATTGGCACAGC-3'; R: 5'-AGTGCTTGCTGAATCATGATGC-3'

Table S2. List of mitochondrial genomes from Spariformes and Perciformes species used in this study, with *Perca fluviatilis* and *Epinephelus coioides* included as the outgroups.

Order	Family	Name	Accession	Length(bp)	
				Total	D-loop
Spariformes	Lethrinidae	***Lethrinus olivaceus***	PV872036	16792	1119
		Lethrinus atlanticus	OQ420716	16789	1059
		Lethrinus laticaudis	NC_030353	16758	1045
		Lethrinus nebulosus	PP897909	16785	1077
		Lethrinus obsoletus	NC_009855	16779	1142
		Monotaxis grandoculis	NC_010957	16174	563
		Gnathodentex aureolineatus	NC_063714	16940	1311
	Nemipteridae	***Pentapodus caninus***	PV872034	16866	1199
		Pentapodus setosus	NC_086456	18943	3269
		Nemipterus bathybius	NC_029938	17353	1669
		Nemipterus furcosus	LC549804	16882	1166
		Nemipterus hexodon	NC_049150	17115	1428
		Nemipterus japonicus	NC_023972	16995	1387
		Nemipterus randalli	NC_062624	16642	960
		Nemipterus virgatus	NC_027734	16992	1327
		Nemipterus balinensoides	NC_082988	17032	1363
		Scolopsis ciliata	NC_043917	16733	1042
		Scolopsis vosmeri	NC_029489	16770	1094
	Sparidae	*Acanthopagrus latus*	NC_056780	16635	1024
		Calamus penna	NC_082991	16599	986
		Dentex dentex	NC_037755	16656	1081
		Dentex gibbosus	NC_037731	16771	1158
		Diplodus holbrookii	NC_082990	16623	1012
		Evynnis tumifrons	NC_029479	16616	1000
		Lagodon rhomboides	OP057024	16688	1082
		Pagellus bogaraveo	NC_009502	16941	1338
		Pagrus auriga	NC_005146	16628	1014
		Pagrus major	NC_003196	17031	1420
		Pagrus pagrus	NC_072936	16685	1071
		Parargyrops edita	NC_008616	16640	1028
		Polysteganus undulosus	NC_084230	16626	1075
		Rhabdosargus sarba	NC_025301	16644	1040
		Sparus aurata	KT805959	16652	1047
Perciformes	Serranidae	*Epinephelus coioides*	NC_011111	16418	791
	Percidae	*Perca fluviatilis*	NC_026313	16537	941

Table S3. Merged partition scheme and best-fit nucleotide substitution models inferred by ModelFinder for mitochondrial protein-coding genes (PCGs) of 35 fishes' species.

**Part**	**Genes (partition set)**	**Sites**	**Best-fit model**
Part 1	*CYTB* (codonA*); *COX2* (codonA); *COX3* (codonA)	869	TIM3e+I+G4
Part 2	*CYTB* (codonB); *COX1* (codonB); *COX2* (codonB); *COX3* (codonB); *ND1* (codonB)	1708	GTR+F+I+R2
Part 3	*ATP6* (codonA); *ATP8* (codonA); *ND2* (codonA); *ND3* (codonA); *ND4* (codonA); *ND5* (codonA)	1809	GTR+F+I+G4
Part 4	*ATP6* (codonB); *ND2* (codonB); *ND3* (codonB); *ND*4*L* (codonB); *ND4* (codonB); *ND5* (codonB)	1852	GTR+F+R3
Part 5	*ATP8* (codonB); *ND6* (codonB)	227	TVM+F+G4
Part 6	*COX1* (codonA)	516	GTR+F+I+G4
Part 7	*ND1* (codonA); *ND4L* (codonA)	421	GTR+F+R3
Part 8	*ND6* (codonA)	172	GTR+F+I+G4

* codonA = 1st+2nd codon positions；codonB = 3rd position.

Table S4. Nucleotide composition of the mitochondrial PCGs of *Pentapodus caninus*.

PCG	Length (bp)	A (%)	T (%)	G (%)	C (%)	A+T (%)	G+C (%)	AT-Skew	GC-Skew
ND1	975	24.8	30.9	14.2	30.2	55.7	44.4	-0.109	-0.361
ND2	1047	26.9	25.7	12.5	34.9	52.6	47.4	0.024	-0.472
COX1	1551	24.3	31.4	18.8	25.5	55.7	44.3	-0.127	-0.15
COX2	691	27.5	28.2	17.4	26.9	55.7	44.3	-0.013	-0.216
ATP8	168	31.5	25.6	13.7	29.2	57.1	42.9	0.104	-0.361
ATP6	684	25.9	30.8	14.3	28.9	56.7	43.2	-0.088	-0.338
COX3	786	25.4	28.4	16.8	29.4	53.8	46.2	-0.054	-0.273
ND3	351	21.4	30.5	17.9	30.2	51.9	48.1	-0.176	-0.254
ND4L	297	20.2	27.6	17.5	34.7	47.8	52.2	-0.155	-0.329
ND4	1381	26.2	28.2	15.8	29.8	54.4	45.6	-0.037	-0.307
ND5	1839	27.9	28.4	14.0	29.6	56.3	43.6	-0.009	-0.357
ND6	522	16.5	40.0	29.5	14.0	56.5	43.5	-0.417	0.357
CYTB	1141	24.6	30.5	16.0	28.8	55.1	44.8	-0.107	-0.285
Total	11430	25.4	29.6	16.3	28.7	55.0	45.0	-0.077	-0.276

Table S5. Nucleotide composition of the mitochondrial PCGs of *Lethrinus olivaceus*.

PCG	Length (bp)	A (%)	T (%)	G (%)	C (%)	A+T (%)	G+C (%)	AT-Skew	GC-Skew
ND1	972	24.2	27.7	15.3	32.8	51.9	48.1	-0.067	-0.363
ND2	1047	24.9	24.9	13.0	37.2	49.8	50.2	0	-0.482
COX1	1551	23.4	29.2	20.1	27.3	52.6	47.4	-0.11	-0.151
COX2	691	27.8	26	16.9	29.2	53.8	46.1	0.032	-0.266
ATP8	168	28.6	26.8	11.9	32.7	55.4	44.6	0.032	-0.467
ATP6	684	21.3	28.2	15.9	34.5	49.5	50.4	-0.139	-0.368
COX3	786	24.2	26.6	17.9	31.3	50.8	49.2	-0.048	-0.271
ND3	351	20.5	31.1	15.1	33.3	51.6	48.4	-0.204	-0.376
ND4L	297	19.2	25.3	18.5	37.0	44.5	55.5	-0.136	-0.333
ND4	1381	25.0	26.7	16.2	32.1	51.7	48.3	-0.034	-0.328
ND5	1839	26.5	27.0	14.6	31.9	53.5	46.5	-0.01	-0.373
ND6	522	16.9	37.5	31.4	14.2	54.4	45.6	-0.38	0.378
CYTB	1141	23.9	29.2	15.3	31.6	53.1	46.9	-0.099	-0.346
Total	11427	24.1	27.9	16.8	31.2	52.0	48.0	-0.072	-0.299

Table S6. Presence or absence of *tRNA-Val* gene duplication in the *12S–tRNA-Val–16S* region across representative Spariformes mitogenomes.

**Order**	**Family**	**Name**	***12S–tRNA-Val–16S*** **region length (bp)**	***tRNA-Val*** **Duplication**
Spariformes	Lethrinidae	***Lethrinus olivaceus***	202	YES*
		Lethrinus atlanticus	74	NO
		Lethrinus laticaudis	76	NO
		Lethrinus nebulosus	165	YES
		Lethrinus obsoletus	170	YES
		Monotaxis grandoculis	72	NO
		Gnathodentex aureolineatus	74	NO
	Nemipteridae	***Pentapodus caninus***	96	NO
		Pentapodus setosus	72	NO
		Nemipterus bathybius	72	NO
		Nemipterus furcosus	72	NO
		Nemipterus hexodon	72	NO
		Nemipterus japonicus	109	NO
		Nemipterus randalli	72	NO
		Nemipterus virgatus	72	NO
		Nemipterus balinensoides	72	NO
		Scolopsis ciliata	72	NO
		Scolopsis vosmeri	72	NO
	Sparidae	*Acanthopagrus latus*	72	NO
		Calamus penna	72	NO
		Dentex dentex	110	NO
		Dentex gibbosus	72	NO
		Diplodus holbrookii	72	NO
		Evynnis tumifrons	72	NO
		Lagodon rhomboides	72	NO
		Pagellus bogaraveo	72	NO
		Pagrus auriga	72	NO
		Pagrus major	72	NO
		Pagrus pagrus	72	NO
		Parargyrops edita	72	NO
		Polysteganus undulosus	73	NO
		Rhabdosargus sarba	72	NO
		Sparus aurata	72	NO

*Note: Region length indicates the sequence from the end of *12S rRNA* to the start of *16S rRNA*, including all tRNA-Val copies and intergenic spacers. YES indicates the presence of tandem *tRNA-Val* gene duplication; NO indicates a single copy.

Table S7. Codon frequencies and relative synonymous codon usage (RSCU) of the mitochondrial PCGs of fish species of Percoidei.

**Codon**	***Lethrinus olivaceus***		*Lethrinus atlanticus*		*Lethrinus laticaudis*		*Lethrinus nebulosus*		*Lethrinus obsoletus*		*Monotaxis grandoculis*		*Gnathodentex aureolineatus*		***Pentapodus caninus***		*Pentapodus setosus*		*Nemipterus bathybius*	
	**Count**	**RSCU**	**Count**	**RSCU**	**Count**	**RSCU**	**Count**	**RSCU**	**Count**	**RSCU**	**Count**	**RSCU**	**Count**	**RSCU**	**Count**	**RSCU**	**Count**	**RSCU**	**Count**	**RSCU**
**UUU (F)***	100	0.85	86	0.75	82	0.72	82	0.72	84	0.72	71	0.64	94	0.82	114	0.93	104	0.84	100	0.84
**UUC (F)**	136	1.15	144	1.25	147	1.28	147	1.28	148	1.28	152	1.36	135	1.18	130	1.07	145	1.16	137	1.16
**UUA (L)**	74	0.67	94	0.85	69	0.62	60	0.55	63	0.57	60	0.54	70	0.63	111	1.06	117	1.11	117	1.14
**UUG (L)**	26	0.24	21	0.19	15	0.14	26	0.24	23	0.21	22	0.2	26	0.23	17	0.16	18	0.17	28	0.27
**UCU (S)**	47	1.14	45	1.13	40	0.99	44	1.09	43	1.07	33	0.8	42	1.01	53	1.4	45	1.13	69	1.68
**UCC (S)**	81	1.97	77	1.93	83	2.05	78	1.93	83	2.06	80	1.94	85	2.04	56	1.48	65	1.63	56	1.37
**UCA (S)**	51	1.24	56	1.41	55	1.36	55	1.36	50	1.24	63	1.53	48	1.15	58	1.53	72	1.81	58	1.41
**UCG (S)**	10	0.24	9	0.23	10	0.25	13	0.32	13	0.32	11	0.27	14	0.34	11	0.29	7	0.18	7	0.17
**UAU (Y)**	37	0.67	37	0.67	42	0.76	36	0.65	42	0.76	35	0.62	35	0.62	51	0.9	48	0.83	44	0.75
**UAC (Y)**	74	1.33	74	1.33	69	1.24	75	1.35	69	1.24	77	1.38	78	1.38	62	1.1	67	1.17	74	1.25
**UGU (C)**	7	0.58	10	0.8	5	0.42	5	0.4	8	0.62	5	0.37	13	0.9	11	0.76	14	1	10	0.65
**UGC (C)**	17	1.42	15	1.2	19	1.58	20	1.6	18	1.38	22	1.63	16	1.1	18	1.24	14	1	21	1.35
**UGA (W)**	93	1.55	100	1.67	93	1.56	101	1.7	94	1.58	103	1.72	95	1.56	91	1.57	99	1.72	102	1.74
**UGG (W)**	27	0.45	20	0.33	26	0.44	18	0.3	25	0.42	17	0.28	27	0.44	25	0.43	16	0.28	15	0.26
**CUU (L)**	161	1.46	156	1.41	143	1.29	142	1.29	143	1.29	108	0.97	156	1.4	160	1.53	163	1.55	142	1.38
**CUC (L)**	165	1.5	177	1.6	198	1.79	196	1.78	195	1.76	232	2.07	212	1.9	133	1.27	103	0.98	130	1.26
**CUA (L)**	166	1.51	162	1.47	181	1.64	178	1.62	176	1.59	178	1.59	148	1.33	159	1.52	199	1.89	152	1.48
**CUG (L)**	68	0.62	52	0.47	58	0.52	58	0.53	65	0.59	71	0.63	57	0.51	48	0.46	33	0.31	48	0.47
**CCU (P)**	54	0.95	51	0.9	67	1.19	60	1.06	58	1.03	43	0.77	40	0.71	66	1.19	75	1.35	83	1.52
**CCC (P)**	111	1.96	104	1.84	97	1.72	103	1.82	106	1.88	94	1.69	97	1.73	81	1.47	74	1.33	63	1.16
**CCA (P)**	51	0.9	57	1.01	51	0.9	46	0.81	49	0.87	73	1.31	67	1.2	63	1.14	62	1.12	57	1.05
**CCG (P)**	11	0.19	14	0.25	11	0.19	17	0.3	13	0.23	13	0.23	20	0.36	11	0.2	11	0.2	15	0.28
**CAU (H)**	37	0.7	35	0.67	35	0.68	30	0.58	21	0.41	16	0.3	20	0.38	33	0.62	34	0.64	31	0.6
**CAC (H)**	68	1.3	69	1.33	68	1.32	73	1.42	82	1.59	90	1.7	86	1.62	73	1.38	73	1.36	73	1.4
**CAA (Q)**	77	1.52	88	1.71	82	1.61	76	1.49	84	1.66	74	1.53	85	1.75	81	1.65	82	1.67	93	1.88
**CAG (Q)**	24	0.48	15	0.29	20	0.39	26	0.51	17	0.34	23	0.47	12	0.25	17	0.35	16	0.33	6	0.12
**CGU (R)**	13	0.67	16	0.83	12	0.62	14	0.73	12	0.62	15	0.78	14	0.74	11	0.56	11	0.56	11	0.55
**CGC (R)**	15	0.77	13	0.68	16	0.82	13	0.68	17	0.88	14	0.73	12	0.63	16	0.82	10	0.51	11	0.55
**CGA (R)**	36	1.85	41	2.13	38	1.95	38	1.97	40	2.08	40	2.08	40	2.11	45	2.31	49	2.48	50	2.5
**CGG (R)**	14	0.72	7	0.36	12	0.62	12	0.62	8	0.42	8	0.42	10	0.53	6	0.31	9	0.46	8	0.4
**AUU (I)**	120	0.9	128	0.96	142	1.05	114	0.85	111	0.85	112	0.84	106	0.81	171	1.21	178	1.33	176	1.27
**AUC (I)**	146	1.1	139	1.04	128	0.95	153	1.15	151	1.15	156	1.16	157	1.19	112	0.79	90	0.67	102	0.73

Codon	***Lethrinus olivaceus***		*Lethrinus atlanticus*		*Lethrinus laticaudis*		*Lethrinus nebulosus*		*Lethrinus obsoletus*		*Monotaxis grandoculis*		*Gnathodentex aureolineatus*		***Pentapodus caninus***		*Pentapodus setosus*		*Nemipterus bathybius*	
	**Count**	**RSCU**	**Count**	**RSCU**	**Count**	**RSCU**	**Count**	**RSCU**	**Count**	**RSCU**	**Count**	**RSCU**	**Count**	**RSCU**	**Count**	**RSCU**	**Count**	**RSCU**	**Count**	**RSCU**
**AUA (M)**	84	1.14	64	0.83	74	0.95	63	0.83	73	0.97	73	1.01	76	1.03	82	1.05	92	1.16	112	1.29
**AUG (M)**	63	0.86	90	1.17	81	1.05	89	1.17	78	1.03	71	0.99	71	0.97	74	0.95	66	0.84	62	0.71
**ACU (T)**	56	0.75	47	0.64	45	0.61	39	0.53	34	0.47	42	0.56	50	0.7	60	0.8	57	0.78	61	0.87
**ACC (T)**	119	1.6	125	1.71	127	1.73	127	1.73	130	1.79	128	1.7	113	1.59	109	1.45	99	1.36	98	1.39
**ACA (T)**	103	1.39	105	1.43	105	1.43	112	1.52	106	1.46	102	1.36	102	1.44	115	1.53	117	1.6	114	1.62
**ACG (T)**	19	0.26	16	0.22	16	0.22	16	0.22	21	0.29	29	0.39	19	0.27	17	0.23	19	0.26	9	0.13
**AAU (N)**	45	0.79	35	0.59	41	0.68	31	0.51	32	0.53	31	0.54	27	0.5	43	0.77	45	0.8	44	0.77
**AAC (N)**	69	1.21	84	1.41	80	1.32	91	1.49	88	1.47	84	1.46	82	1.5	69	1.23	67	1.2	70	1.23
**AAA (K)**	61	1.67	59	1.62	66	1.78	61	1.67	61	1.63	61	1.74	59	1.69	64	1.75	63	1.73	62	1.68
**AAG (K)**	12	0.33	14	0.38	8	0.22	12	0.33	14	0.37	9	0.26	11	0.31	9	0.25	10	0.27	12	0.32
**AGU (S)**	9	0.22	8	0.2	9	0.22	9	0.22	9	0.22	11	0.27	12	0.29	13	0.34	13	0.33	16	0.39
**AGC (S)**	49	1.19	44	1.1	46	1.14	43	1.07	44	1.09	49	1.19	49	1.18	36	0.95	37	0.93	40	0.98
**GUU (V)**	62	1.11	58	1.03	57	1.05	69	1.21	60	1.07	51	0.91	66	1.13	76	1.28	67	1.13	81	1.3
**GUC (V)**	70	1.25	70	1.24	77	1.41	76	1.33	80	1.43	80	1.43	89	1.52	56	0.94	56	0.94	67	1.07
**GUA (V)**	61	1.09	71	1.26	59	1.08	53	0.93	50	0.89	55	0.99	50	0.85	88	1.48	97	1.63	80	1.28
**GUG (V)**	31	0.55	26	0.46	25	0.46	30	0.53	34	0.61	37	0.66	29	0.5	18	0.3	18	0.3	22	0.35
**GCU (A)**	72	0.82	59	0.67	72	0.82	65	0.73	69	0.76	59	0.65	57	0.62	84	0.94	82	0.92	80	0.92
**GCC (A)**	161	1.83	167	1.9	159	1.8	170	1.92	164	1.82	173	1.92	180	1.95	130	1.45	134	1.51	126	1.45
**GCA (A)**	101	1.15	113	1.28	108	1.22	102	1.15	109	1.21	107	1.19	116	1.25	124	1.39	128	1.44	123	1.41
**GCG (A)**	18	0.2	13	0.15	14	0.16	17	0.19	19	0.21	22	0.24	17	0.18	20	0.22	11	0.12	19	0.22
**GAU (D)**	17	0.44	20	0.51	17	0.45	15	0.39	14	0.37	16	0.42	16	0.43	30	0.77	20	0.51	24	0.64
**GAC (D)**	61	1.56	58	1.49	58	1.55	61	1.61	62	1.63	60	1.58	58	1.57	48	1.23	58	1.49	51	1.36
**GAA (E)**	72	1.47	78	1.56	60	1.22	68	1.39	65	1.35	74	1.44	60	1.2	74	1.48	74	1.51	77	1.62
**GAG (E)**	26	0.53	22	0.44	38	0.78	30	0.61	31	0.65	29	0.56	40	0.8	26	0.52	24	0.49	18	0.38
**GGU (G)**	26	0.43	42	0.7	22	0.37	31	0.51	24	0.4	25	0.42	17	0.29	42	0.7	34	0.56	24	0.4
**GGC (G)**	83	1.38	76	1.26	92	1.53	84	1.39	90	1.49	92	1.56	110	1.87	94	1.56	89	1.47	88	1.45
**GGA (G)**	77	1.28	77	1.28	65	1.08	71	1.18	78	1.29	63	1.07	61	1.04	71	1.18	80	1.32	81	1.33
**GGG (G)**	55	0.91	46	0.76	62	1.03	55	0.91	49	0.81	56	0.95	47	0.8	34	0.56	40	0.66	50	0.82

Codon	*Nemipterus balinensoides*		*Nemipterus randalli*		*Nemipterus hexodon*		*Nemipterus virgatus*		*Nemipterus japonicus*		*Nemipterus furcosus*		*Scolopsis ciliata*		*Scolopsis vosmeri*		*Epinephelus coioides*		*Perca fluviatilis*	
	**Count**	**RSCU**	**Count**	**RSCU**	**Count**	**RSCU**	**Count**	**RSCU**	**Count**	**RSCU**	**Count**	**RSCU**	**Count**	**RSCU**	**Count**	**RSCU**	**Count**	**RSCU**	**Count**	**RSCU**
**UUU (F)**	101	0.87	97	0.84	92	0.8	102	0.89	102	0.89	73	0.63	101	0.83	112	0.96	113	0.94	124	1.06
**UUC (F)**	130	1.13	135	1.16	139	1.2	127	1.11	127	1.11	159	1.37	141	1.17	122	1.04	127	1.06	111	0.94
**UUA (L)**	125	1.2	133	1.29	119	1.15	138	1.32	138	1.32	71	0.68	83	0.76	110	1.02	113	1.03	110	0.99
**UUG (L)**	23	0.22	22	0.21	29	0.28	20	0.19	20	0.19	28	0.27	25	0.23	25	0.23	14	0.13	21	0.19
**UCU (S)**	63	1.53	70	1.71	57	1.41	61	1.56	61	1.56	48	1.19	49	1.2	63	1.54	44	1.09	47	1.2
**UCC (S)**	57	1.38	55	1.35	61	1.51	58	1.48	58	1.48	70	1.73	69	1.68	57	1.39	71	1.76	67	1.71
**UCA (S)**	55	1.34	56	1.37	58	1.44	54	1.38	54	1.38	60	1.48	63	1.54	57	1.39	64	1.59	58	1.48
**UCG (S)**	9	0.22	10	0.24	8	0.2	8	0.2	8	0.2	13	0.32	10	0.24	15	0.37	7	0.17	8	0.2
**UAU (Y)**	67	1.13	39	0.66	40	0.67	50	0.81	52	0.83	38	0.62	46	0.79	62	1.02	45	0.8	56	0.98
**UAC (Y)**	52	0.87	79	1.34	80	1.33	74	1.19	73	1.17	84	1.38	71	1.21	60	0.98	67	1.2	58	1.02
**UGU (C)**	12	0.77	9	0.58	14	0.88	18	1.12	18	1.12	15	0.86	13	0.79	16	0.97	13	0.84	9	0.78
**UGC (C)**	19	1.23	22	1.42	18	1.12	14	0.88	14	0.88	20	1.14	20	1.21	17	1.03	18	1.16	14	1.22
**UGA (W)**	92	1.55	104	1.75	102	1.71	106	1.78	106	1.78	97	1.63	103	1.76	101	1.7	105	1.75	106	1.77
**UGG (W)**	27	0.45	15	0.25	17	0.29	13	0.22	13	0.22	22	0.37	14	0.24	18	0.3	15	0.25	14	0.23
**CUU (L)**	147	1.41	150	1.46	147	1.42	134	1.28	134	1.28	141	1.34	177	1.63	184	1.71	141	1.28	198	1.78
**CUC (L)**	110	1.06	106	1.03	112	1.08	131	1.25	131	1.25	174	1.66	157	1.44	141	1.31	153	1.39	119	1.07
**CUA (L)**	158	1.52	166	1.61	184	1.77	162	1.55	162	1.55	163	1.55	146	1.34	144	1.34	203	1.85	171	1.54
**CUG (L)**	61	0.59	41	0.4	31	0.3	43	0.41	42	0.4	52	0.5	64	0.59	42	0.39	35	0.32	48	0.43
**CCU (P)**	66	1.21	83	1.54	69	1.27	66	1.21	65	1.19	64	1.2	65	1.16	79	1.39	57	1.05	87	1.57
**CCC (P)**	85	1.55	64	1.19	72	1.32	82	1.5	82	1.5	83	1.55	82	1.46	68	1.19	100	1.83	83	1.5
**CCA (P)**	57	1.04	59	1.09	63	1.16	59	1.08	60	1.1	54	1.01	56	1	66	1.16	58	1.06	40	0.72
**CCG (P)**	11	0.2	10	0.19	14	0.26	11	0.2	11	0.2	13	0.24	21	0.38	15	0.26	3	0.06	11	0.2
**CAU (H)**	43	0.83	39	0.72	36	0.67	30	0.57	31	0.58	33	0.65	36	0.67	45	0.87	30	0.57	39	0.74
**CAC (H)**	61	1.17	69	1.28	71	1.33	75	1.43	75	1.42	69	1.35	71	1.33	59	1.13	76	1.43	66	1.26
**CAA (Q)**	86	1.83	87	1.81	88	1.85	89	1.84	89	1.84	82	1.71	71	1.53	84	1.81	82	1.73	84	1.66
**CAG (Q)**	8	0.17	9	0.19	7	0.15	8	0.16	8	0.16	14	0.29	22	0.47	9	0.19	13	0.27	17	0.34
**CGU (R)**	5	0.26	7	0.35	11	0.56	9	0.46	9	0.46	9	0.45	12	0.62	16	0.82	14	0.71	15	0.79
**CGC (R)**	15	0.77	12	0.6	13	0.66	11	0.56	11	0.56	12	0.6	18	0.92	12	0.62	16	0.81	13	0.68
**CGA (R)**	46	2.36	49	2.45	51	2.58	46	2.33	46	2.33	56	2.8	39	2	45	2.31	43	2.18	40	2.11
**CGG (R)**	12	0.62	12	0.6	4	0.2	13	0.66	13	0.66	3	0.15	9	0.46	5	0.26	6	0.3	8	0.42
**AUU (I)**	192	1.35	187	1.3	176	1.2	189	1.35	187	1.34	149	1.05	153	1.09	180	1.32	158	1.14	173	1.33
**AUC (I)**	92	0.65	100	0.7	118	0.8	91	0.65	93	0.66	135	0.95	127	0.91	92	0.68	119	0.86	88	0.67
**AUA (M)**	106	1.25	110	1.26	118	1.39	122	1.39	122	1.38	98	1.2	90	1.18	91	1.17	108	1.4	99	1.35
**AUG (M)**	64	0.75	65	0.74	52	0.61	54	0.61	55	0.62	65	0.8	62	0.82	65	0.83	46	0.6	48	0.65

Codon	*Nemipterus balinensoides*		*Nemipterus randalli*		*Nemipterus hexodon*		*Nemipterus virgatus*		*Nemipterus japonicus*		*Nemipterus furcosus*		*Scolopsis ciliata*		*Scolopsis vosmeri*		*Epinephelus coioides*		*Perca fluviatilis*	
	**Count**	**RSCU**	**Count**	**RSCU**	**Count**	**RSCU**	**Count**	**RSCU**	**Count**	**RSCU**	**Count**	**RSCU**	**Count**	**RSCU**	**Count**	**RSCU**	**Count**	**RSCU**	**Count**	**RSCU**
**ACU (T)**	78	1.06	67	0.91	61	0.82	61	0.85	61	0.85	59	0.79	48	0.69	63	0.9	51	0.65	56	0.76
**ACC (T)**	92	1.26	100	1.37	104	1.4	101	1.41	100	1.4	104	1.39	96	1.38	81	1.16	126	1.62	120	1.63
**ACA (T)**	106	1.45	118	1.61	121	1.63	111	1.55	112	1.57	126	1.69	111	1.59	109	1.56	132	1.69	104	1.41
**ACG (T)**	17	0.23	8	0.11	11	0.15	14	0.2	13	0.18	10	0.13	24	0.34	27	0.39	3	0.04	15	0.2
**AAU (N)**	53	0.96	48	0.83	42	0.76	47	0.84	46	0.82	36	0.68	55	0.98	41	0.75	33	0.57	45	0.78
**AAC (N)**	57	1.04	67	1.17	69	1.24	65	1.16	66	1.18	70	1.32	57	1.02	68	1.25	82	1.43	70	1.22
**AAA (K)**	63	1.66	67	1.79	64	1.73	62	1.68	62	1.68	60	1.62	60	1.6	65	1.67	72	1.87	67	1.79
**AAG (K)**	13	0.34	8	0.21	10	0.27	12	0.32	12	0.32	14	0.38	15	0.4	13	0.33	5	0.13	8	0.21
**AGU (S)**	17	0.41	15	0.37	18	0.45	17	0.43	17	0.43	14	0.35	14	0.34	17	0.41	10	0.25	17	0.43
**AGC (S)**	46	1.12	39	0.96	40	0.99	37	0.94	37	0.94	38	0.94	41	1	37	0.9	46	1.14	38	0.97
**GUU (V)**	70	1.17	65	1.1	69	1.19	79	1.33	79	1.33	75	1.23	69	1.24	85	1.42	56	1.06	88	1.53
**GUC (V)**	59	0.98	67	1.14	56	0.97	50	0.84	50	0.84	69	1.14	63	1.13	63	1.05	56	1.06	61	1.06
**GUA (V)**	83	1.38	78	1.32	83	1.44	90	1.52	89	1.5	77	1.27	65	1.17	67	1.12	77	1.45	64	1.11
**GUG (V)**	28	0.47	26	0.44	23	0.4	18	0.3	19	0.32	22	0.36	26	0.47	24	0.4	23	0.43	17	0.3
**GCU (A)**	92	1.06	82	0.96	78	0.91	88	1.01	88	1.02	64	0.75	75	0.85	87	1	62	0.73	77	0.86
**GCC (A)**	114	1.32	120	1.41	116	1.36	113	1.3	112	1.29	141	1.64	141	1.59	122	1.4	146	1.73	158	1.77
**GCA (A)**	132	1.53	113	1.33	127	1.49	128	1.48	128	1.48	116	1.35	122	1.38	118	1.35	121	1.43	105	1.17
**GCG (A)**	8	0.09	26	0.3	20	0.23	18	0.21	18	0.21	22	0.26	16	0.18	22	0.25	9	0.11	18	0.2
**GAU (D)**	28	0.74	29	0.73	31	0.79	29	0.74	29	0.74	19	0.45	28	0.72	33	0.82	16	0.41	25	0.65
**GAC (D)**	48	1.26	50	1.27	47	1.21	49	1.26	49	1.26	65	1.55	50	1.28	47	1.18	62	1.59	52	1.35
**GAA (E)**	76	1.57	78	1.7	81	1.71	77	1.6	77	1.6	69	1.48	63	1.27	77	1.56	80	1.65	71	1.42
**GAG (E)**	21	0.43	14	0.3	14	0.29	19	0.4	19	0.4	24	0.52	36	0.73	22	0.44	17	0.35	29	0.58
**GGU (G)**	42	0.69	29	0.48	37	0.61	25	0.41	25	0.4	33	0.55	33	0.55	45	0.75	39	0.66	47	0.77
**GGC (G)**	73	1.21	87	1.43	76	1.25	84	1.37	85	1.38	73	1.22	100	1.65	79	1.32	90	1.52	77	1.26
**GGA (G)**	76	1.26	82	1.34	98	1.61	87	1.41	87	1.41	88	1.47	68	1.12	70	1.17	76	1.28	72	1.18
**GGG (G)**	51	0.84	46	0.75	33	0.54	50	0.81	50	0.81	46	0.77	41	0.68	45	0.75	32	0.54	49	0.8

*Note: A = Ala, F = Phe, C = Cys, D = Asp, N = Asn, E = Glu, Q = Gln, G = Gly, H = His, L = Leu, I = Ile, K = Lys, M = Met, P = Pro, R = Arg, S = Ser, T = Thr, V = Val, W = Trp, Y = Tyr
